# Nanopore Data-Driven Chromosome-Level Assembly of Flax Genome

**DOI:** 10.3390/plants13243465

**Published:** 2024-12-11

**Authors:** Alexander A. Arkhipov, Elena N. Pushkova, Nadezhda L. Bolsheva, Tatiana A. Rozhmina, Elena V. Borkhert, Daiana A. Zhernova, Tatiana Yu. Rybakova, Nikolai M. Barsukov, Olesya D. Moskalenko, Elizaveta A. Sigova, Ekaterina M. Dvorianinova, Nataliya V. Melnikova, Alexey A. Dmitriev

**Affiliations:** 1 Engelhardt Institute of Molecular Biology, Russian Academy of Sciences, 119991 Moscow, Russia; arkhipov.aleksandr2.0@gmail.com (A.A.A.); pushkova18@gmail.com (E.N.P.); nlbolsheva@mail.ru (N.L.B.); sashai@inbox.ru (E.V.B.); zhernova.d@ya.ru (D.A.Z.); tatyana.rybakova.04@mail.ru (T.Y.R.); keepter@yandex.ru (N.M.B.); moskalenko.od@phystech.edu (O.D.M.); sigova.ea@phystech.edu (E.A.S.); dvorianinova.em@phystech.edu (E.M.D.); 2 Federal Research Center for Bast Fiber Crops, 172002 Torzhok, Russia; tatyana_rozhmina@mail.ru; 3 Faculty of Biology, Lomonosov Moscow State University, 119234 Moscow, Russia; 4 Russian State Agrarian University—Moscow Timiryazev Agricultural Academy, 127434 Moscow, Russia; 5 Moscow Institute of Physics and Technology, 141701 Moscow, Russia

**Keywords:** flax, *Linum usitatissimum*, nanopore sequencing, HERRO, Hifiasm, Verkko, genome assembly, T2T chromosomes

## Abstract

Flax is an important crop grown for seed and fiber. Flax chromosome number is 2n = 30, and its genome size is about 450–480 Mb. To date, the genomes of several flax varieties have been sequenced and assembled. However, the obtained assemblies are still far from the telomere-to-telomere (T2T) level. We sequenced the genome of flax variety K-3018 on the Oxford Nanopore Technologies (ONT) platform and obtained 57.7 Gb of R10 simplex reads with an N50 = 18.4 kb (~120× genome coverage). ONT reads longer than 50 kb were kept as ultra-long ones (~10× genome coverage), and the rest of the ONT reads were corrected using the HERRO R10 model (quality > Q10, length > 10 kb, ~60× genome coverage remained). The genome was assembled using Hifiasm and Verkko. The Hifiasm-generated assembly was 489.1 Mb in length with 54 contigs and an N50 = 28.1 Mb. Verkko produced a very similar but more fragmented genome: 489.1 Mb, 134 contigs, N50 = 17.4 Mb. In the assembly by Hifiasm, eight chromosomes consisted of a single contig with telomeric repeats at both ends. In addition, five chromosomes comprised two contigs and two chromosomes comprised three contigs. These chromosomes also had telomeric repeats at their ends. The Hifiasm-generated assembly of variety K-3018 had similar contiguity but was likely more complete and accurate than the main fifteen-chromosome assembly of variety YY5 (produced from PacBio data and scaffolded with Hi-C data), the most contiguous flax genome assembly at the time of this writing. We suggest that sufficient genome coverage with long ONT R10 simplex reads is a viable alternative to PacBio plus Hi-C data for a high-precision T2T genome assembly of flax, opening new perspectives for whole-genome studies of flax.

## 1. Introduction

Flax (*Linum usitatissimum* L.) is an important crop grown for seed and fiber production [1]. Today, the range of uses of flax is continually expanding. For example, the seeds are valuable for nutraceuticals and pharmaceuticals [2,3,4], while the fiber is promising in the production of composites in the automotive, aerospace, and construction industries [5,6,7,8]. Expanding the uses of flax requires the development of varieties with diverse traits. Data on flax genetics are needed to make this process more efficient.

*L. usitatissimum* has a relatively small genome size (about 450–480 Mb [9,10,11]) and chromosome number 2n = 30 [12,13]. The first genome assembly of flax was obtained in 2012 by Wang et al. [14] for the variety CDC Bethune using the Illumina platform (NCBI Genome, GCA_000224295.1). The size of the assembled genome was 302 Mb with an N50 = 0.69 Mb for scaffolds and an N50 = 0.02 Mb for contigs. Six years later, the CDC Bethune genome was improved to the chromosome level by You et al. [15] using optical (BioNano), BAC-based, and genetic mapping (NCBI Genome, GCA_000224295.2). Each of the fifteen chromosome pseudomolecules consisted of 8 to 23 BioNano contigs with a total size of 316 Mb. In 2020, Zhang et al. [16] obtained three more genome assemblies from 294 to 306 Mb in size with contig N50 values from 0.06 to 0.16 Mb and scaffold N50 values from 0.38 to 1.24 Mb for Longya-10, Heiya-14 (NCBI Genome, GCA_010665265.1), and pale flax (NCBI Genome, GCA_010665285.1) using Illumina sequencing. The Longya-10 genome was then scaffolded to the chromosome level using the Hi-C method and genetic mapping data (NCBI Genome, GCA_010665275.2).

Advances in sequencing technologies allowed the generation of long reads on third-generation sequencing platforms, such as Oxford Nanopore Technologies (ONT) and Pacific Biosciences (PacBio) [17,18,19,20]. In 2021, we assembled the genome of flax variety Atlant (NCBI Genome, GCA_014858635.1) using the ONT and Illumina platforms [21]. The genome assembly size was 362 Mb (N50 = 0.35 Mb), which was larger than the flax genome assemblies obtained earlier with only short Illumina reads. The use of PacBio sequencing allowed Sa et al., in 2021 [9], to assemble the genome of flax variety YY5 (https://zenodo.org/record/4872894, accessed on 15 October 2024) with a size of 455 Mb and an N50 = 9.6 Mb (336 contigs), while Hi-C data brought the assembly to the chromosome level. Thus, the YY5 genome assembly was significantly more extended than previously assembled flax genomes. In 2022, we assembled the genome of flax variety 3896 (NCBI Genome, GCA_030674075.2) based on ONT reads with further polishing using Illumina reads [10,22]. The genome assembly size of 3896 (447 Mb) was close to that of YY5 and was characterized by a high N50 of contigs—6.2 Mb. In 2023, we obtained another flax genome (variety K-1531) with high characteristics: genome assembly size = 413 Mb and N50 = 5.2 Mb [23]. In 2023, Zhao et al. [11] applied PacBio sequencing, Hi-C technology, and genetic mapping to produce a chromosome-level assembly of Neiya No. 9 (https://zenodo.org/records/7811972, accessed on 15 October 2024), which had a size of 474 Mb with a contig N50 = 0.9 Mb. Therefore, the use of third-generation sequencing enabled the generation of more complete and extended flax genomes compared to the second-generation sequencing platforms, such as Illumina, which was mainly associated with a better assembly of genomic repeats through the use of long reads [10]. However, the obtained flax genomes are still far from the telomere-to-telomere (T2T) level, which is becoming the new standard for plants [20]. Gapless T2T plant genomes allow us to identify segmental duplications containing genes that may be misassembled in lower-quality genomes, assemble and completely characterize missing transposable elements, and reveal repeat regions containing important regulatory elements [19,24].

For obtaining the flax genome assemblies from ONT R9 simplex reads, the Canu assembler showed the best results [21,23]. The Hifiasm and MaSuRCA assemblers were successfully used to generate flax genomes from PacBio data [9,11]. At the same time, genome assemblers are advancing along with sequencing technologies, and software applications are emerging to improve the accuracy of sequencing data. In the present study, we aimed to obtain a high-quality assembly of the flax genome and get closer to the T2T level using ONT R10 simplex reads and state-of-the-art bioinformatics tools.

## 2. Results

As a result of the DNA sequencing of flax variety K-3018 on the ONT platform, 57.7 Gb of R10 simplex reads with an N50 = 18.4 kb were obtained, corresponding to ~120× genome coverage (with an expected genome length of ~480 Mb, selected based on the longest flax genome assembly obtained for Neiya No. 9 [11]). Reads from 10 to 50 kb (36.3 Gb) were then processed using the HERRO R10 model, resulting in 29.3 Gb of corrected reads (~60× genome coverage). In addition, 5.0 Gb of reads longer than 50 kb were selected as ultra-long (~10× genome coverage). Corrected reads from 10 to 50 kb and uncorrected ultra-long reads were used for further genome assembly.

Assembly of the K-3018 genome was performed using Hifiasm and Verkko. Hifiasm produced a genome of 489.13 Mb consisting of 54 contigs with an N50 = 28.08 Mb (Table 1). Verkko assembled a more fragmented genome of 489.08 Mb with 134 contigs and an N50 = 17.41 Mb. Meanwhile, the contig-level assembly of variety YY5 from PacBio HiFi data, which is probably the best genome of flax in terms of contiguity at the time of this writing, consists of 336 contigs. Scaffolding based on Hi-C data and alignment to the genome assembly v2 of flax variety CDC Bethune as a reference allowed Sa et al. to reduce the number of contigs to 15 long (corresponding to fifteen chromosomes) and 246 short (five of which were longer than 1 Mb) [9]. Thus, both the Hifiasm- and Verkko-generated assemblies of the K-3018 genome were characterized by a better N50 value than the YY5 genome assembly. Both K-3018 assemblies were also longer (489.1 Mb each) than the YY5 assembly (455.0 Mb) (Table 1).

**Figure 1:**
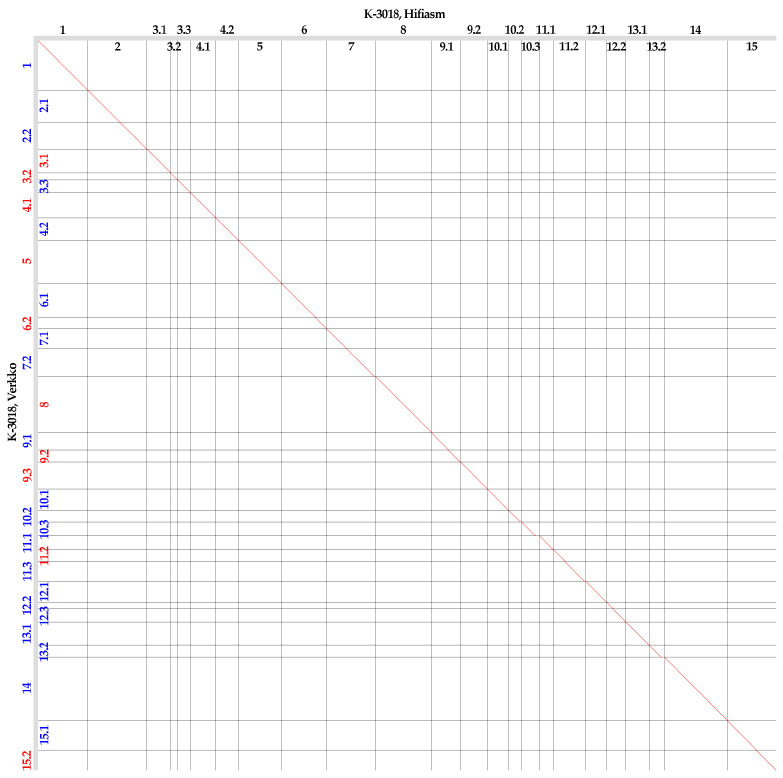
Global alignment of the genome assemblies of flax variety K-3018 generated by Verkko (Y axis) and Hifiasm (Y axis). Contigs longer than 3 Mb are presented. Red numbers—forward contigs, blue numbers—reverse contigs (relative to the Hifiasm-produced assembly).

**Figure 2:**
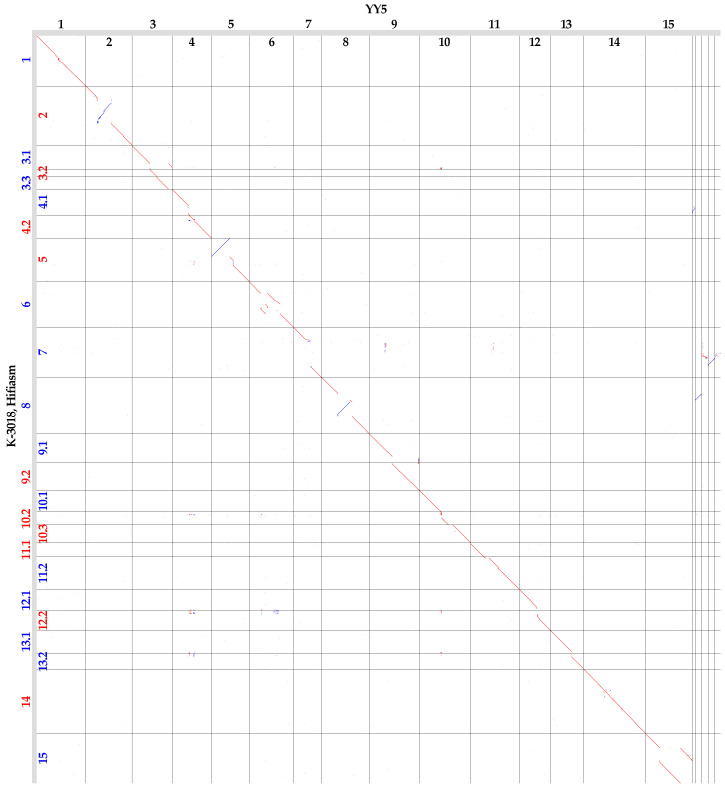
Global alignment of the genome assemblies of flax varieties K-3018 (Hifiasm, Y axis) and YY5 (X axis). Contigs longer than 3 Mb are presented for K-3018 and contigs longer than 1 Mb are presented for YY5. Red numbers—forward contigs, blue numbers—reverse contigs (relative to the YY5 genome assembly).

We compared the K-3018 genome assemblies (generated by Hifiasm and Verkko) with each other (Figure 1) and with the YY5 assembly (Figure 2). Contigs in the K-3018 assemblies were named according to the chromosome numbers of the flax variety YY5, which are identical to those of CDC Bethune.

Global genome alignments showed that Hifiasm and Verkko produced similar genome assemblies, but the assembly by Hifiasm was more contiguous than the assembly by Verkko (Figure 1).

The Hifiasm-generated genome assembly of K-3018 was relatively similar to the YY5 genome assembly (Figure 2). However, some notable differences were identified, such as translocations and inversions in chromosomes 2, 5, 6, 8, and 15. In addition, gaps in chromosomes 7 and 8 were revealed in the YY5 assembly, which may be due to the more complete assembly of K-3018, as evidenced by the longer assembly length. Furthermore, some of the YY5 contigs that were longer than 1 Mb but were not scaffolded into the fifteen chromosomes were located in the gaps. This also suggested some errors in the YY5 assembly. Thus, the Hifiasm-generated assembly of K-3018 based on ONT R10 simplex data was slightly more fragmented than the scaffolded assembly of YY5 but significantly outperformed it at the contig level and included chromosome regions that were missing in the fifteen chromosomes of YY5.

To further assess the quality of the Hifiasm- and Verkko-generated genome assemblies of K-3018, a search for telomeric repeats was performed. In the assembly by Hifiasm, the TTTAGGG repeats (corresponding to telomeres) were identified at both ends of eight contigs (1, 2, 5, 6, 7, 8, 14, and 15) (Supplementary Figure S1). These contigs likely represent T2T chromosomes 1, 2, 5, 6, 7, 8, 14, and 15. In addition, telomeric repeats were identified at one end of ten contigs (4.1 and 4.2, 9.1 and 9.2, 11.1 and 11.2, 12.1 and 12.2, and 13.1 and 13.2), probably corresponding to five whole chromosomes (4, 9, 11, 12, and 13) split into two contigs. Chromosomes 3 and 10 likely consisted of three contigs, two of which (3.1 and 3.3, 10.1 and 10.3) contained telomeric repeats at one of their ends. Thus, all fifteen flax chromosomes contained telomeric repeats at their ends in the K-3018 genome assembly produced by Hifiasm.

In the Verkko-generated genome assembly of K-3018, telomeric repeats were identified at both ends of four contigs corresponding to chromosomes 1, 5, 8, and 14, and at one end of sixteen contigs corresponding to two parts of chromosomes 2, 3, 4, 6, 11, 12, 13, and 15 (Supplementary Figure S2). For chromosome 7, we failed to identify telomeric repeats at the end of contig 7.2. For chromosomes 9 and 10, assembly errors were possibly present in contigs 9.2 and 10.2—telomeric repeats were identified at their ends, but the contigs were in the middle of chromosomes 9 and 10, respectively (both chromosomes consisted of three contigs; see Figure 1). Thus, Hifiasm also outperformed Verkko in the assembly of telomeric and centromeric repeats.

It should be noted that chromosome 14 in the Hifiasm- and Verkko-generated assemblies of K-3018 had extended TTTAGGG telomeric repeats not only at the ends but also in the internal part of the chromosome (Supplementary Figures S1 and S2). Furthermore, such a structure of chromosome 14 was also characteristic of the YY5 genome assembly (Supplementary Figure S3). This fact requires further studies, although we have previously identified a large interstitial locus of telomeric repeats in the centromeric heterochromatin of a pair of the largest chromosomes of *L. usitatissimum* using FISH [25].

**Figure 3:**
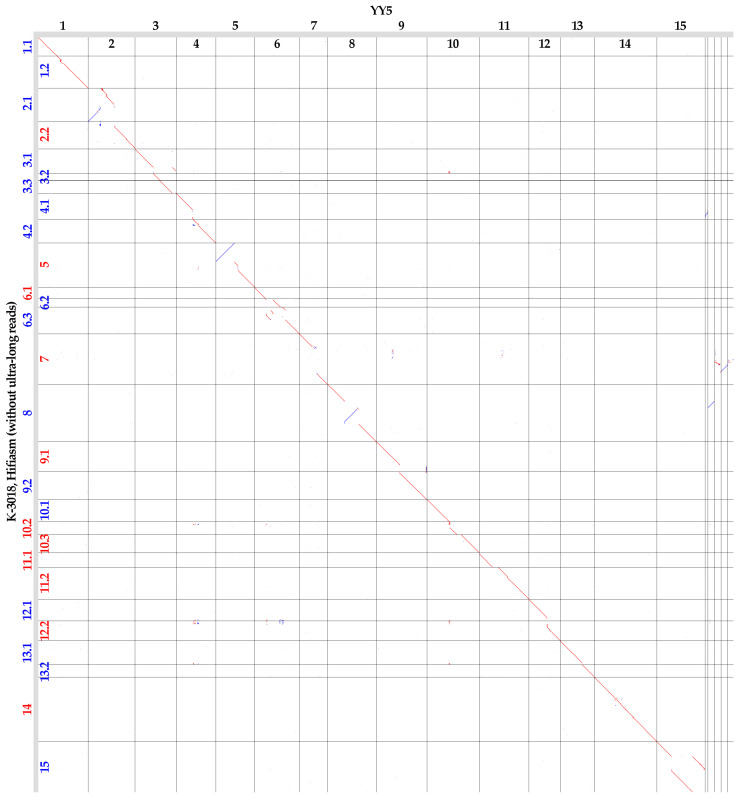
Global alignment of the genome assemblies of flax varieties K-3018 (Hifiasm without ultra-long reads, Y axis) and YY5 (X axis). Contigs longer than 3 Mb are presented for K-3018 and contigs longer than 1 Mb are presented for YY5. Red numbers—forward contigs, blue numbers—reverse contigs (relative to the YY5 genome assembly).

Chromosome 5 in the Hifiasm-generated genome assembly of K-3018 had a long inversion compared to the YY5 assembly (Figure 2). However, telomeric repeats were identified at both ends of chromosome 5 of K-3018 (Supplementary Figure S1), suggesting the correctness of its assembly, whereas telomeric repeats were absent at one end of chromosome 5 of YY5 (Supplementary Figure S3). Chromosomes 6 and 15 of K-3018 had translocations compared to the YY5 assembly. Chromosome 6 of YY5 lacked telomeric repeats at both ends (Supplementary Figure S3). Chromosome 15 of YY5 also did not have telomeric repeats at the ends, but they were detected in the middle of the chromosome (Supplementary Figure S3). At the same time, telomeric repeats were localized at the correct positions of chromosomes 6 and 15 in the Hifiasm-generated assembly of K-3018, specifically at the ends. We proposed that these three large chromosomal rearrangements were errors in the YY5 genome assembly. The same may be true for the large inversions in chromosomes 2 and 8 (Figure 2), but this cannot be verified by the location of telomeric repeats. We did not use scaffolding but performed assembly from long ONT reads, which reduces the possibility of incorrect ordering of contig parts during assembly. As an additional check of the Hifiasm-generated assembly for errors in the orientation of long contigs, we obtained the K-3018 genome assembly without the use of ultra-long reads and aligned it to the YY5 assembly (Figure 3). The K-3018 assembly produced by Hifiasm without ultra-long reads became more fragmented, but all five large rearrangements relative to the YY5 assembly remained. Further studies are needed to elucidate the status of these rearrangements, but we suggest that the errors in the YY5 genome assembly are more likely.

Finally, we assessed the completeness of the Hifiasm- and Verkko-generated genome assemblies of K-3018 using BUSCOs (Benchmarking Universal Single Copy Orthologs). Both assemblies had 2228 out of 2326 complete BUSCOs, corresponding to a completeness score of 95.79% based on the eudicots_odb10 dataset (Table 2). In both K-3018 assemblies, the percentage of duplicated BUSCOs was slightly higher than in the YY5 assembly.

To assess the accuracy of the genome assembly produced by Hifiasm, we aligned the transcriptome data for leaves of K-3018, previously obtained by us on the Illumina platform [26], to the K-3018 assembly. The number of genomic sites covered five or more times by the transcriptome data was calculated and the variant allele frequencies (VAF) for these sites were evaluated. Illumina reads are highly accurate, and if an alternative nucleotide was present at a particular site with a high VAF (>30%), we concluded that there was an error in the genome assembly. In the Hifiasm-generated assembly of K-3018, 99.986% of the covered genomic sites had the same nucleotide as in the transcriptome data, which is a high value. The same analysis was performed for the YY5 genome assembly and the Illumina leaf transcriptome reads used for its annotation [9]. In the YY5 genome assembly, the nucleotides in 99.873% of the covered genomic sites were the same as in the transcriptome data. Thus, we concluded that the obtained sequence of the K-3018 genome was of high accuracy.

## 3. Discussion

Using the ONT R10 sequencing data corrected with HERRO for genome assembly by Hifiasm, we obtained the high-quality assembly of the flax variety K-3018 genome, in which eight chromosomes consisted of a single contig, five chromosomes consisted of two contigs, and two chromosomes consisted of three contigs. This assembly was significantly more contiguous than the assemblies we previously obtained for flax using ONT R9 data: the N50 value for K-3018 was 28.1 Mb compared to 0.35 Mb for Atlant [16], 6.2 Mb for 3896 [18], and 5.2 Mb for K-1531 [19]. In the case of Atlant, a relatively small volume of ONT data was obtained with an R9.4.1 MinION flow cell (8.4 Gb), which negatively affected assembly contiguity. In the case of 3896, the ONT data volume was higher (30 Gb from R9.4.1 flow cells), which improved assembly contiguity and allowed us to obtain an N50 in a Mb range. In the case of K-1531, the data volume was 23 Gb, which was not very high, but one-third of the data were obtained with a precision R10.4.1 MinION flow cell, likely contributing to an assembly contiguity, which was similar to that of 3896. Several bioinformatics tools were tested for the assembly of the above-mentioned flax genomes, and Canu [27] always gave the best results. However, Canu requires a lot of computational time, which grows exponentially with the increase in the ONT data volume.

In the present work, we generated significantly more ONT data (57.7 Gb) than in our previous studies on the flax genome and used a precision R10.4.1 PromethION flow cell. This allowed us to apply a different approach to genome assembly. First, the ONT data were processed with HERRO [28] to obtain corrected reads with improved accuracy. The accuracy of the corrected reads was sufficient for them to be used for assembly with fast T2T assemblers: Hifiasm [29] and Verkko [30]. These assemblers were originally developed to work with accurate third-generation sequencing data, mainly PacBio HiFi reads, and were efficiently used to assemble T2T genomes [30,31,32,33,34,35,36,37,38]. However, the development of R10.4.1 flow cells and correction tools such as HERRO opened the possibility of applying such assemblers to obtain high-quality genomes from ONT simplex data [28,38,39]. Such an approach to flax genome assembly yielded great results. Fifteen flax chromosomes were represented by twenty-four Hifiasm-assembled contigs without scaffolding with Hi-C or Pore-C data. The genome assembly produced by Verkko was more fragmented, but Hifiasm- and Verkko-generated contigs aligned well. Overall, the metrics of the assembly by Hifiasm outperformed those of the assembly by Verkko, although the main difference was in the degree of fragmentation.

The assemblies of the K-3018 genome obtained from ONT simplex reads with Hifiasm and Verkko were longer than that of the YY5 genome generated from PacBio reads with Hifiasm and further scaffolded to the chromosome level with Hi-C data and the CDC Bethune genome assembly v2 as reference: 489 Mb vs. 455 Mb [17]. This may be related to the long lengths of the ONT reads, which allow for more accurate assembly of long repetitive genomic regions [40]. Indeed, the K-3018 assembly was consistent with the most contiguous (at the time of this writing) flax genome assembly of variety YY5; however, we identified some large chromosomal differences, including long genomic regions in chromosomes 7 and 8 that were absent in the YY5 assembly but present in the K-3018 assembly. In the expanded assembly of the YY5 genome (261 contigs), we identified contigs longer than 1 Mb that corresponded to the gaps but were not included in the main fifteen-chromosome assembly of YY5. Therefore, the YY5 genome may be slightly underassembled compared to the K-3018 genome. In addition, the comparison of the K-3018 and YY5 assemblies revealed several long inversions and translocations. This may be due to differences between the varieties, but most likely it is a result of errors in the YY5 genome assembly. In chromosomes with such rearrangements, telomeric repeats were located at the ends in the K-3018 assembly but were missing or located in the middle in the YY5 assembly. However, the contributions of genotype and genome assembly approach will be more fully resolved as more flax genomes with T2T chromosomes are assembled.

In addition, we evaluated the sequence accuracy of the K-3018 genome assembly by aligning the K-3018 Illumina transcriptome data to it. We showed that the error rate was only 0.014% for the genomic sites covered five or more times by the transcriptome data and that the K-3018 assembly was superior to the YY5 assembly in this parameter. PacBio HiFi reads are indeed more accurate than ONT reads [19]. However, read correction tools such as HERRO (50-fold reduction in errors in ONT reads across multiple datasets [28]) and further use of efficient genome assemblers such as Hifiasm make it possible to obtain highly accurate genome assemblies using only ONT R10 simplex sequencing data.

Thus, the HERRO read correction allowed the use of Hifiasm with ONT R10 simplex data to assemble the flax genome with similar contiguity to the flax genome obtained from PacBio reads and scaffolded with Hi-C data. In addition, the genome assembled from the ONT data was likely more complete and accurate. Furthermore, ONT sequencing is currently significantly less expensive than PacBio sequencing. We suggest that sufficient genome coverage with long ONT R10 simplex reads is a viable cost-effective alternative to PacBio plus Hi-C data for high-precision T2T assemblies of flax genomes. This approach opens new perspectives for broad research on the genetic diversity of flax varieties at the whole-genome level and for the construction of a flax pan-genome. Moreover, the existence of the contiguous and accurate genome assembly may significantly improve the results of quantitative trait locus (QTL) and quantitative trait nucleotide (QTN) identification, since most of such studies in flax used the CDC Bethune genome assembly as a reference [41,42,43], in which a substantial part of the genome was lost and the error rate was quite high. In addition, the K-3018 genome assembly is valuable for studying gene families with many duplicated genes, which was a problem with the low-quality flax genome assemblies. Such studies are necessary for efficient identification of genetic determinants of valuable flax traits, genome editing, and the development of improved varieties based on marker-assisted and genomic selection.

## 4. Materials and Methods

### 4.1. Plant Material

A typical plant of the intermediate flax variety K-3018 (collection of the Institute for Flax, Torzhok, Russia) was selected and seeds were obtained from it. The name of K-3018 is Ozimi lan; it originates from Yugoslavia. This genotype is part of our set of varieties for flax pan-genome construction, which covers flax diversity based on origin, phenotype, and genetic relationship data [26]. Seeds were sterilized in 1% NaClO solution and planted in fifteen-liter pots with soil for 4 weeks. Then, the tops of the plant shoots were covered with dark cloth (this step is necessary to reduce the metabolite content in the leaves and increase DNA purity). After 7 days, leaves under dark cloth were collected in 2 mL tubes and immediately frozen in liquid nitrogen. Leaves were stored at −70 °C until DNA isolation.

### 4.2. DNA Extraction and Sequencing

DNA was extracted as previously described, including isolation of nuclei [10]. DNA concentration and quality were assessed using a NanoDrop spectrophotometer (Thermo Fisher Scientific, Waltham, MA, USA), a Qubit fluorometer (Thermo Fisher Scientific), and gel electrophoresis (0.5% agarose, 30 h at 25 V). The SQK-LSK114 kit (Oxford Nanopore Technologies, Oxford, UK) was used for DNA library preparation. Genome sequencing was performed on a PromethION sequencer (Oxford Nanopore Technologies) with an R10.4.1 flow cell. The generated ONT R10 simplex data were basecalled using Dorado v.7.2.13 (https://github.com/nanoporetech/dorado, accessed on 15 October 2024) with the super accuracy model and minimum quality of Q7. The obtained ONT reads were deposited in the NCBI SRA: SRX26506354.

### 4.3. Data Correction and Genome Assembly

ONT R10 simplex data correction was performed using the HERRO algorithm [28] integrated into the Dorado correct module. Reads with the following criteria were corrected: average quality of Q10 or higher; read length between 10,000 and 50,000 nucleotides. Reads longer than 50,000 nucleotides were not corrected and were kept as ultra-long reads for use in hybrid assembly.

Flax is a self-pollinated plant, so the copies of its chromosomes (diploid, 2n = 30) are nearly identical. Because of this fact, the “-l0” option was used to disable the haplotig duplicate purging stage for the assembly by Hifiasm v.0.19.9-r616 [29]. For the assembly by Verkko v.2.1 [30], the “--haploid” option was used. Both assemblies were performed in hybrid data mode, combining corrected simplex reads with ultra-long uncorrected simplex reads.

### 4.4. Genome Assembly Quality Assessment

Assembly statistics were obtained using QUAST v.5.2.0 [44]. Assembly completeness was evaluated with BUSCO v.5.7.1 [45] using the eudicots_odb10 dataset. Telomeric repeats (TTTAGGG) at the ends of potential chromosomes were identified with Tidk v.0.2.63 [46]. Contigs generated by Hifiasm and Verkko for the K-3018 genome were filtered for length (>3 Mb) and aligned to each other and to the YY5 genome assembly (contigs longer than 1 Mb were considered) using LAST v.1584 [47].

### 4.5. Variant Allele Frequency Analysis

VAF analysis was performed using PPLine [48]. First, the transcriptome data for leaves of K-3018 (NCBI SRA, SRR26620385) [26] were filtered using trimmomatic v. 0.39 [49] with the following parameters: TRAILING:28, SLIDINGWINDOW:4:24, MINLEN:40. The transcriptome data were then aligned to the K-3018 genome assembly using BWA-MEM v.0.7.17-r1188 [50] with the following parameters: -k 15 -A 1 -B 1 -O 1 -E 1 -L 0 -w 500 -r 1.2 -c 10000. Variant calling was performed with FreeBayes v1.3.2 [51] with the following parameters: --min-mapping-quality 10 --min-base-quality 12 --min-supporting-allele-qsum 10 --min-alternate-total 4 --min-coverage 5 --min-alternate-fraction 0.05. The same analysis was performed for the leaf transcriptome data used to annotate the YY5 genome assembly (NCBI SRA, SRX10695182) and the YY5 genome assembly as reference (https://zenodo.org/record/4872894, accessed on 15 October 2024) [9]. Only forward transcriptome reads trimmed to 76 bp were used for YY5 to standardize them with the K-3018 transcriptome reads.

## Figures and Tables

**Table 1:** Statistics of genome assemblies of flax varieties K-3018 and YY5.

Assembly	N50, Mb	L50	Assembly Length	Contigs
K-3018 (Hifiasm)	28.08	8	489.13 Mb	54
K-3018 (Verkko)	17.41	10	489.08 Mb	134
YY5 (contigs)	9.61	-	454.95 Mb	336
YY5 (scaffolds)	30.52	7		15 *

* A total of 246 additional relatively short contigs (5 of which were longer than 1 Mb) were also generated.

**Table 2:** BUSCO statistics of the genome assemblies of flax varieties K-3018 and YY5.

Assembly	Complete	Single Copy	Duplicated	Fragmented	Missing
K-3018, Hifiasm	95.79%	21.67%	74.12%	0.64%	3.57%
K-3018, Verkko	95.79%	21.54%	74.25%	0.64%	3.57%
YY5	95.79%	22.23%	73.56%	0.60%	3.61%

## Data Availability

The data presented in this study are available in the NCBI Sequence Read Archive (SRA) under the BioProject accession number PRJNA648016.

## Supplementary Materials

The following supporting information can be downloaded at: https://www.mdpi.com/article/10.3390/plants13243465/s1, Figure S1: Telomeric repeats in the Hifiasm-generated genome assembly of flax variety K-3018; Figure S2: Telomeric repeats in the Verkko-generated genome assembly of flax variety K-3018; Figure S3: Telomeric repeats in the genome assembly of flax variety YY5.
