# Supplementary material for: Nanopore Data-Driven Chromosome-Level Assembly of Flax Genome
Source: Plants (Basel). 2024 Dec 11;13(24):3465. doi: 10.3390/plants13243465 (PMC11679535; doi:10.3390/plants13243465)

Supplementary Figure S1. Telomeric repeats in the Hifiasm-generated genome assembly of flax variety K-3018.

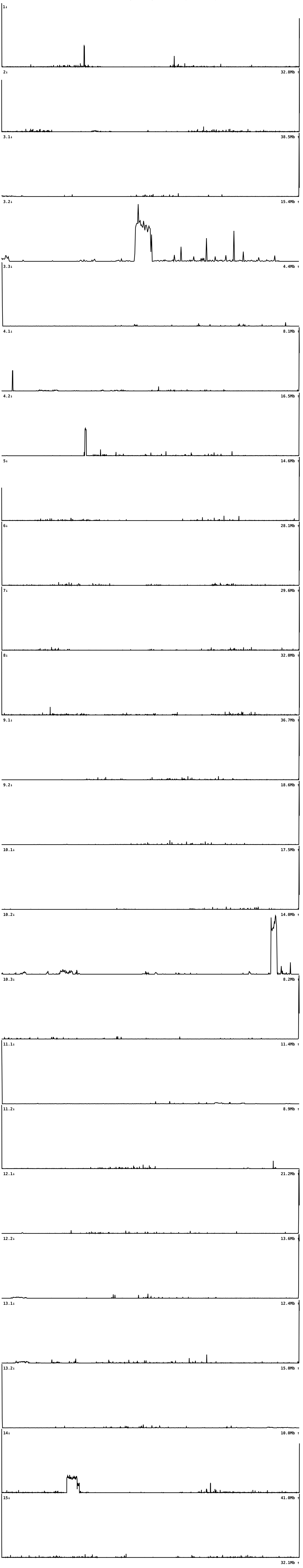

Supplement: Supplementary file 1 [file plants-13-03465-s001.zip › Figure_S1_Hifiasm_2024.12.02.pdf]
