# Supplementary material for: Nanopore Data-Driven Chromosome-Level Assembly of Flax Genome
Source: Plants (Basel). 2024 Dec 11;13(24):3465. doi: 10.3390/plants13243465 (PMC11679535; doi:10.3390/plants13243465)

**Supplementary Figure S2.** Telomeric repeats in the Verkko-generated genome assembly of flax variety K- 3018.

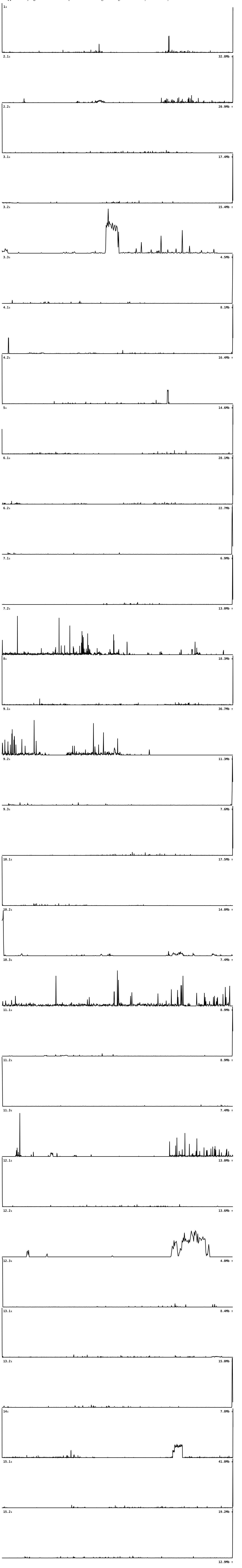

Supplement: Supplementary file 1 [file plants-13-03465-s001.zip › Figure_S2_Verkko_2024.12.02.pdf]
